# Post-Stroke Administration of L-4F Promotes Neurovascular and White Matter Remodeling in Type-2 Diabetic Stroke Mice

**DOI:** 10.3389/fneur.2022.863934

**Published:** 2022-04-28

**Authors:** Min Zhou, Rongwen Li, Poornima Venkat, Yu Qian, Michael Chopp, Alex Zacharek, Julie Landschoot-Ward, Brianna Powell, Quan Jiang, Xu Cui

**Affiliations:** ^1^Department of Neurology, Henry Ford Hospital, Detroit, MI, United States; ^2^Department of Physics, Oakland University, Rochester, MI, United States

**Keywords:** diabetes, stroke, blood–brain barrier (BBB), white matter (WM), ABCA1, HDL, neuroinflammation

## Abstract

Patients with type 2 diabetes mellitus (T2DM) exhibit a distinct and high risk of ischemic stroke with worse post-stroke neurovascular and white matter (WM) prognosis than the non-diabetic population. In the central nervous system, the ATP-binding cassette transporter member A 1 (ABCA1), a reverse cholesterol transporter that efflux cellular cholesterol, plays an important role in high-density lipoprotein (HDL) biogenesis and in maintaining neurovascular stability and WM integrity. Our previous study shows that L-4F, an economical apolipoprotein A member I (ApoA-I) mimetic peptide, has neuroprotective effects *via* alleviating neurovascular and WM impairments in the brain of db/db-T2DM stroke mice. To further investigate whether L-4F has neurorestorative benefits in the ischemic brain after stroke in T2DM and elucidate the underlying molecular mechanisms, we subjected middle-aged, brain-ABCA1 deficient (ABCA1^−B/−B^), and ABCA1-floxed (ABCA1^fl/fl^) T2DM control mice to distal middle cerebral artery occlusion. L-4F (16 mg/kg, subcutaneous) treatment was initiated 24 h after stroke and administered once daily for 21 days. Treatment of T2DM-stroke with L-4F improved neurological functional outcome, and decreased hemorrhage, mortality, and BBB leakage identified by decreased albumin infiltration and increased tight-junction and astrocyte end-feet densities, increased cerebral arteriole diameter and smooth muscle cell number, and increased WM density and oligodendrogenesis in the ischemic brain in both ABCA1^−B/−B^ and ABCA1^fl/fl^ T2DM-stroke mice compared with vehicle-control mice, respectively (*p* < 0.05, *n* = 9 or 21/group). The L-4F treatment reduced macrophage infiltration and neuroinflammation identified by decreases in ED-1, monocyte chemoattractant protein-1 (MCP-1), and toll-like receptor 4 (TLR4) expression, and increases in anti-inflammatory factor Insulin-like growth factor 1 (IGF-1) and its receptor IGF-1 receptor β (IGF-1Rβ) in the ischemic brain (*p* < 0.05, *n* = 6/group). These results suggest that post-stroke administration of L-4F may provide a restorative strategy for T2DM-stroke by promoting neurovascular and WM remodeling. Reducing neuroinflammation in the injured brain may contribute at least partially to the restorative effects of L-4F independent of the ABCA1 signaling pathway.

## Introduction

Type-2 diabetes (T2DM) constitutes ~90% of all diabetic patients and is a major risk factor for ischemic and hemorrhagic stroke. Epidemiological investigation in both younger (15–49 years) (1) and elderly (>65 years) (2) diabetic patients show a 10 years cumulative recurrent ischemic stroke rate of 29.7% for T2DM, and 12.0% for non-diabetic patients in the 15–49 age group (1), and 4.26% of stroke events and 1.79% of death rates during 6 years of follow-up for >65 age group. Moreover, diabetic-stroke patients exhibit a high level of neuroinflammation, worse neurovascular and white matter (WM) injury with severe and long-lasting neurological deficits compared with non-diabetic stroke patients (1–6). Neuroinflammation is involved in the onset and progression of stroke which is triggered by the infiltration of blood macrophages (M1 macrophage) and the activation of glial cells (microglia and astrocytes) which then released proinflammatory cytokines/factors. These inflammatory mediators not only lead to neurotoxicity and neuronal dysfunction and also induce blood–brain barrier (BBB) disruption and leakage. BBB damage allows the trafficking of immune cells and/or plasma proteins into the brain parenchyma which amplifies neuroinflammation and exacerbates neurovascular and WM injury (7). Therefore, there is a compelling need to develop therapeutic strategies for T2DM-stroke patients and elucidate the underlying molecular biological mechanisms.

After adjusting for all possible covariables, blood levels of low-density lipoprotein (LDL) cholesterol show a significant association with increased risk of stroke and mortality (2, 8). However, high-density lipoprotein (HDL) and HDL-increasing agents, such as Niacin, GW3965, T090317, etc., have demonstrated anti-neuroinflammation capabilities (9–14) and protection of brain vasculature and WM after stroke in preclinical studies (15–20). In T2DM patients, levels of both HDL and apolipoproteins and their functions such as the antioxidative capacity are impaired, which contribute to neurovascular and WM damage after stroke (8–12, 21–25).

In humans, the circulating blood contains only about 40% of the total amount of HDL, and most of the HDL in the central nervous system (CNS) is synthesized *in situ* mainly by the ATP-binding cassette transporter member A 1 (ABCA1), a reverse cholesterol transporter that efflux cellular cholesterol (26, 27). ABCA1 not only plays a key role in HDL biogenesis and in maintaining brain cholesterol metabolism, but it also exerts highly anti-atherogenic effects and has remarkable anti-inflammatory properties (28–33). We have previously demonstrated that deficiency of ABCA1 in the brain induces worse neurological functional deficits after stroke, increases BBB leakage and aggravates OL loss and WM injury (17, 34–36). The Apolipoprotein A-I (ApoA-I) mimetic peptide, 4F (D-4F, synthesized from D-amino acids, and L-4F, synthesized from L-amino acids) increases cholesterol efflux (37–43) and has anti-inflammatory effects (44–46). In our previous study, we have shown that treatment of stroke in type 1 diabetes mellitus (T1DM) rats with D-4F or db/db-T2DM stroke mice treated with L-4F significantly decreases neurovascular and WM damage and improves neurological function in the early stage (4–7 days) after stroke (45, 46). However, whether L-4F is capable of crossing the BBB and whether long-term post-stroke treatment with L-4F promotes neurovascular and WM remodeling and improves recovery of neurological function in T2DM, and whether ABCA1 mediates L-4F-induced neurorestoration have not been studied. Therefore, in this study, we employ middle-aged, brain-ABCA1 deficient (ABCA1^−B/−B^) and ABCA1-floxed (ABCA1^fl/fl^) control mice that were induced with T2DM and subjected to stroke, to test whether L-4F treatment initiated at 24 h after onset of ischemic stroke enhances neurological recovery. We also test if L-4F treatment improves vascular and WM rewiring in T2DM stroke and whether L-4F decreases inflammation *via* ABCA1 dependent signaling pathway. We demonstrate that L-4F can pass through the BBB and has neurorestorative capabilities in promoting neurovascular and WM remodeling, and oligodendrogenesis in the ischemic brain of T2DM-stroke mice. Our data also indicate that reducing neuroinflammation in the injured brain may contribute at least partially to the neurorestorative effects of L-4F independent of the ABCA1 signaling pathway.

## Materials and Methods

### T2DM Induction

To investigate whether the ABCA1 signaling pathway mediates L-4F-induced neurorestorative in T2DM-stroke mice, ABCA1^−B/−B^ and ABCA1^fl/fl^ mice were employed. The original breeders were provided by Dr. Michael Hayden from the University of British Columbia, Canada. All mice used in this study were self-bred in the Bioresources of Henry Ford Health System, and all procedures were approved by the Institutional Animal Care and Use Committee of Henry Ford Health System (Code No. 1289, Approval Date 04/05, 2021). The litters were heterozygous and the ABCA1^−B/−B^ or ABCA1^fl/fl^ phenotype was identified with genotyping assay (29, 47). While studying the effects of L-4F in both males and females is critical from a translational point of view, in this study, to exclude the effects of estrogen in females and in order to compare results with our previous studies, only male ABCA1^−B/−B^ and ABCA1^fl/fl^ mice (13-month-old, total 115 mice) were employed. These mice were induced with T2DM using a combination of a high-fat diet (HFD, 60% calories from fat, Research Diets, USA) and a low dose of streptozotocin (STZ, ALX-380-010-G001, Enzo Life Science, USA) injection (48–52). Briefly, 10-months-old mice were fed an HFD for 2 months. Then, mice were fasted for 6 h and administered STZ on two consecutive days (75 mg/kg, i.p. as first dose and 50 mg/kg, i.p as the second dose). Mice were continued on an HFD for one more month. Bodyweight and fasting blood glucose levels were measured, and mice with glucose >250 mg/dl were considered as T2DM and included in the study.

### Stroke Model and Treatment

T2DM mice were subjected to permanent distal right middle cerebral artery occlusion (dMCAo) surgery, as described previously (31). At 24 h after dMCAo, mice were randomly divided into 4 groups: 1. ABCA1^fl/fl^ control; 2. ABCA1^fl/fl^ + L-4F; 3. ABCA1^−B/−B^ control; 4. ABCA1^−B/−B^ + L-4F. Mice were administered 16 mg/kg L-4F (BioMatik, Cambridge, ON, Canada) or saline subcutaneously starting at 24 h after dMCAo and once daily for 21 days. The dose of L-4F employed in this study is the same as the optimal dose of D-4F which was employed in our previous study (18). The treatment route using subcutaneous administration of saline or L-4F in this study is consistent with the treatment route employed in our previous publication (46). Mice were sacrificed at 21 days after dMCAo. A total of 86 mice were included excluding mice that died at early time points after stroke and mice with lesion volume <6%. To record mortality, all the animals were counted daily and the percentage of dead animals in each group was counted within the 21 days after dMCAo.

### Behavioral Testing

To evaluate neurological function after stroke, all animals were subject to an adhesive removal test and left foot-fault test before dMCAo (baseline) and on days 1, 3, 7, 14, and 21 after dMCAo, as described previously (19, 53). Experimental groups and treatments were double-blinded to the investigators who performed testing and analyzed data.

### Blood Biochemistry Measurement

To test blood biochemistry, animals were fasted for 6 h, and blood was collected from a tail vein before treatment on day 1 after dMCAo as the baseline measurement and before sacrifice on day 21 after treatment. Blood glucose levels were measured using glucose test strips and a glucose analyzer (Accu-Chek Compact System; Roche Diagnostics, Basel, Switzerland). Blood levels of HDL, total cholesterol (T-CH), and triglyceride were measured using a CardioChek Plus analyzer 2,700 (Polymer Technology System Inc., Indianapolis, IN) and test strips, following the manufacturer's instructions. Each sample was tested in triplicate and the data are presented as mg/dl.

### Cerebral Hemorrhagic Transformation and Lesion Volume Measurement

Briefly, brains were immersion fixed in 4% paraformaldehyde, paraffin-embedded, and then cut into seven (1 mm thick) coronal blocks. A series of 6 μm thick sections were prepared and stained with hematoxylin and eosin (HE), and hemorrhagic transformation was identified by blood cell infiltration in the cerebral ischemic brain and the percentage of hemorrhagic transformation volume was calculated. For lesion volume measurement, the percentage of the indirect lesion volume was calculated, in which the intact area of the ipsilateral hemisphere was subtracted from the area of the contralateral hemisphere, with a micro-computer imaging device (MCID) imaging analysis system (Imaging Research, ST. Catharines, ON, Canada) (54).

### Histochemical and Immunohistological Staining

For histochemical/immunostaining, a standard paraffin block was obtained from the center of the lesion (bregma −1 mm to + 1 mm). A series of 6-μm thick sections were cut from the block. Histochemical-staining for Bielshowsky silver (BS, an axon marker) and Luxol fast blue (LFB, a myelin marker), and histoimmuno-staining for antibodies against albumin (BBB leakage marker, 1:500; ab53435, Abcam), Occludin (a marker of tight junction, OC-3F10, 1:100, Fisher), Aquaporin-4 (AQP4, a protein expressed in astrocytic end-feet as another marker of BBB, 1:1,500; ab3594, EMD Millipore), von Willebrand Factor (vWF, a vessel marker, 1:400; A0082, Dako), α-smooth muscle actin (αSMA, a smooth muscle cell-SMC marker, 1:800, Dako), SMI31 (a marker of phosphorylated-neurofilament, 1:1,000, Covance), adenomatous polyposis coli [APC, a marker of mature oligodendrocytes-OLs (55), Ab-1, OP44, 1:100; Calbiochem], and platelet-derived growth factor receptor alpha (PDGFRα, a marker of oligodendrocyte progenitor cells-OPCs, C-20, SC-338, 1:100, Chemicon) were performed.

Both D-4F and L-4F have robust anti-inflammatory properties (56–58), improve HDL function (56, 59–63), and increase cholesterol efflux (37, 38). In this study, to identify whether D-4F/L-4F can pass the BBB and enter the brain, two ABCA1^fl/fl^-T2DM-stroke mice were subcutaneously administered saline or commercially available FITC-D-4F (Cat#SP160428, Lot#P160412-LR051957, BioMatik, Cambridge, ON, Canada) at 24 and 48 h after dMCAo. Mice were sacrificed 4 h after last treatment and vibratome coronal sections (100 μm) were prepared for immunostaining with vWF, APC, and neuronal nuclei (NeuN, for neurons, 1:300, MAB 377, Chemicon). Images were analyzed using laser scanning confocal microscopy.

For immunostaining measurement, each section containing 8 fields of view within the cortex and corpus callosum (CC) from the ischemic boundary zone (IBZ) were digitized using a 40× objective (Olympus BX40) using a 3-CCD color video camera (Sony DXC-970MD) interfaced with MCID. The percentage of positively stained area for albumin, Occludin, AQP-4, BS, LFB, and SMI-31 was measured using a built-in densitometry function (MCID) with a uniform threshold set above unstained for all the groups and the number of vWF^+^-vessels, αSMA^+^-arterioles, APC^+^-OLs, PDGFR^+^-OPCs and the perimeter of vessels or diameter of arterioles as well as the number of αSMA^+^-SMCs was measured in both the contralateral and the ipsilateral brain.

### Quantification of Myelination on Electronic Microscope Images

To evaluate the ultrastructure of axonal myelination, brain tissues were isolated from the IBZ of CC and processed to ultrathin sections for EM analysis (*n* = 6 mice/group). Axonal structural changes were identified at a magnification of 14,000× in a total of 6 fields of view per animal. The analysis included measurement of the percentage of myelinated axons, thickness of myelin sheath, and G ratio (axon diameter/axon wrapped with myelin diameter ×100%) in 10 ultrathin sections, as described previously (36).

### Western Blot and Real-Time Quantitative PCR (RT-PCR) Assay

Tissue from the ischemic area of the ipsilateral hemisphere from both vehicle-control and L-4F-treated T2DM stroke mice were isolated at 21 days after dMCAo. Brain-tissue lysate was subjected to WB analysis, as described previously (46, 64). The following primary antibodies were used: AQP4 (1:5,000, Milipore, cat# ab3594), myelin basic protein (MBP, 1:500, Millipore, cat# MAB386), CD68 (ED-1, a marker of M1-macrophages, 1:1,000, Serotec, cat# MCA341R), monocyte chemoattractant protein-1 (MCP-1, 1:1,000, Abcam, cat# ab7202), toll-like receptor 4 (TLR4, 1:500, Santa Cruz, cat# sc-10741), Insulin-like growth factor 1 (IGF-1, 1:500, Santa Cruz, cat# sc-9013), IGF-1 receptor β (TGF-1Rβ,1:500, Santa Cruz, cat# sc-713), and β-actin (1:10,000, Abcam, cat# ab6276).

Total RNA was isolated using a standard protocol and quantitative RT-PCR was performed on an ABI Prism 7,000 Sequence Detection System using the Power SYBR Green PCR Master Mix (Applied Biosystems, Foster City, CA). For each sample, the cDNA was generated and used to amplify GAPDH, AQP4, MBP, ED1, MCP-1, TLR4, IGF1, and IGF-1R as described previously (46). All the primers for the RT-PCR assay were designed using Primer Express software (ABI). Each sample was detected in triplicates.

### Statistical Analysis

A total of two-way ANOVA followed by Tukey *post hoc* Test were used for analysis involving four groups, i.e., ABCA1^fl/fl^-T2DM and ABCA1^−B/−B^-T2DM stroke with or without L-4F treatment for analysis blood biochemistry, hemorrhage, mortality, lesion volume, functional outcome, immunostaining, EM measurement, WB and RT-PCR measurement. *p* < 0.05 was set as a significant difference, and all data are presented as mean ± standard error (SE).

In this study, 9 survival animals in each group were employed for the measurement of hemorrhage and lesion volume, and histochemical and immunohisto-staining measurements. The number of animals employed was determined using a priori power calculation; 9 survival stroke animals per group provided 80% power at a significance level of <0.05, assuming a 20% difference in both mean and SD at the 95% CI and a two-sided test. For WB, RT-PCR, and EM analysis, 6 stroke mice per group were needed. Plus, two additional ABCA1^fl/fl^-T2DM-stroke mice, in this study, a total of 86 T2DM-stroke mice were used.

## Results

### L-4F-Treatment Decreases Blood Glucose, Cerebral Hemorrhage, and Mortality, Increases HDL and Improves Functional Outcome After Stroke in T2DM Mice

**Figure 1:**
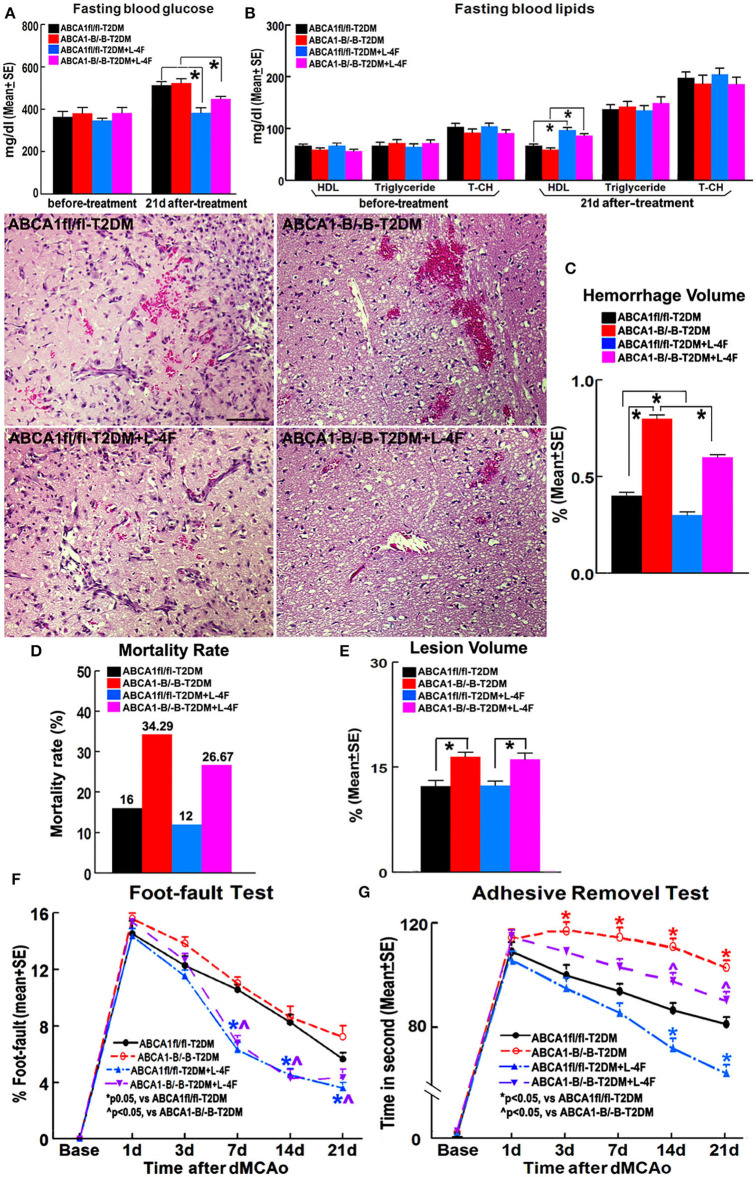
L-4F treatment does not change blood glucose levels before treatment and T-CH and triglyceride after treatment. However, L-4F treatment significantly reduces glucose and increases HDL in the blood 21 days after treatment. L-4F treatment does not alter lesion volume but reduces cerebral hemorrhage and mortality rate as well as improves functional outcome compared with vehicle-control groups in both ABCA1^−B/−B^ and ABCA1^fl/fl^ T2DM-stroke mice, respectively. **A,B** Fasting blood levels of glucose **(A)** and lipids **(B)** measured before L-4F treatment at 1 day after dMCAo and 21 days after treatment. *p* < 0.05, *n* = 21/group; **C–E** Cerebral hemorrhage volume **(C)**, mortality **(D)**, and lesion volume **(E)** at 21 days after L-4F treatment. Scale bar = 100 μm; *p* < 0.05, *n* = 9/group; **F,G**. Foot-fault **(F)** and adhesive removal **(G)** tests before dMCAo and 1, 3, 7, 14, and 21 days after dMCAo. *p < 0.05, *n* = 21/group.

Figure 1 shows that there were no significant differences in the blood levels of glucose before treatment and triglyceride and T-CH at 21 days after treatment among the 4 groups of animals treated with or without L-4F for 21 days after dMCAo. However, L-4F treatment significantly increased blood HDL level and decreased glucose (*p* < 0.05, *n* = 21/group). ABCA1^−B/−B^-T2DM mice exhibit increased hemorrhage, lesion volume, and mortality compared to ABCA1^fl/fl^-T2DM-stroke mice (*p* < 0.05, *n* = 9/group). There were no changes in lesion volume at 21 days after L-4F treatment in both the ABCA1^−B/−B^-T2DM and the ABCA1^fl/fl^-T2DM-stroke mice. However, the hemorrhage volume and mortality rate was dramatically reduced in L-4F treatment groups in both ABCA1^fl/fl^ and ABCA1^−B/−B^ T2DM-stroke mice when compared to the vehicle control group, respectively (*p* < 0.05).

ABCA1^−B/−B^-T2DM stroke mice exhibited significant functional deficits as indicated by increased adhesive-removal time on 3, 7, 14, and 21 days after dMCAo compared with ABCA1^fl/fl^-T2DM mice (*p* < 0.05, *n* = 21/group). L-4F treatment significantly reduced adhesive-removal time at 14 and 21 days after stroke indicating improved sensorimotor function and decreased the left foot-fault rate at 7, 14, and 21 days after treatment indicating an improved motor function in both ABCA1^fl/fl^ and ABCA1^−B/−B^ T2DM-stroke mice (*p* < 0.05, *n* = 21/group). These data indicate that L-4F treatment increases blood HDL and reduces glucose levels, decreases cerebral hemorrhagic transformation and mortality as well as improves neurological functional outcomes in T2DM-stroke mice.

### L-4F Treatment Decreases BBB Leakage and Cerebral Vascular Damage in the Ischemic Brain of T2DM-Stroke Mice

**Figure 2:**
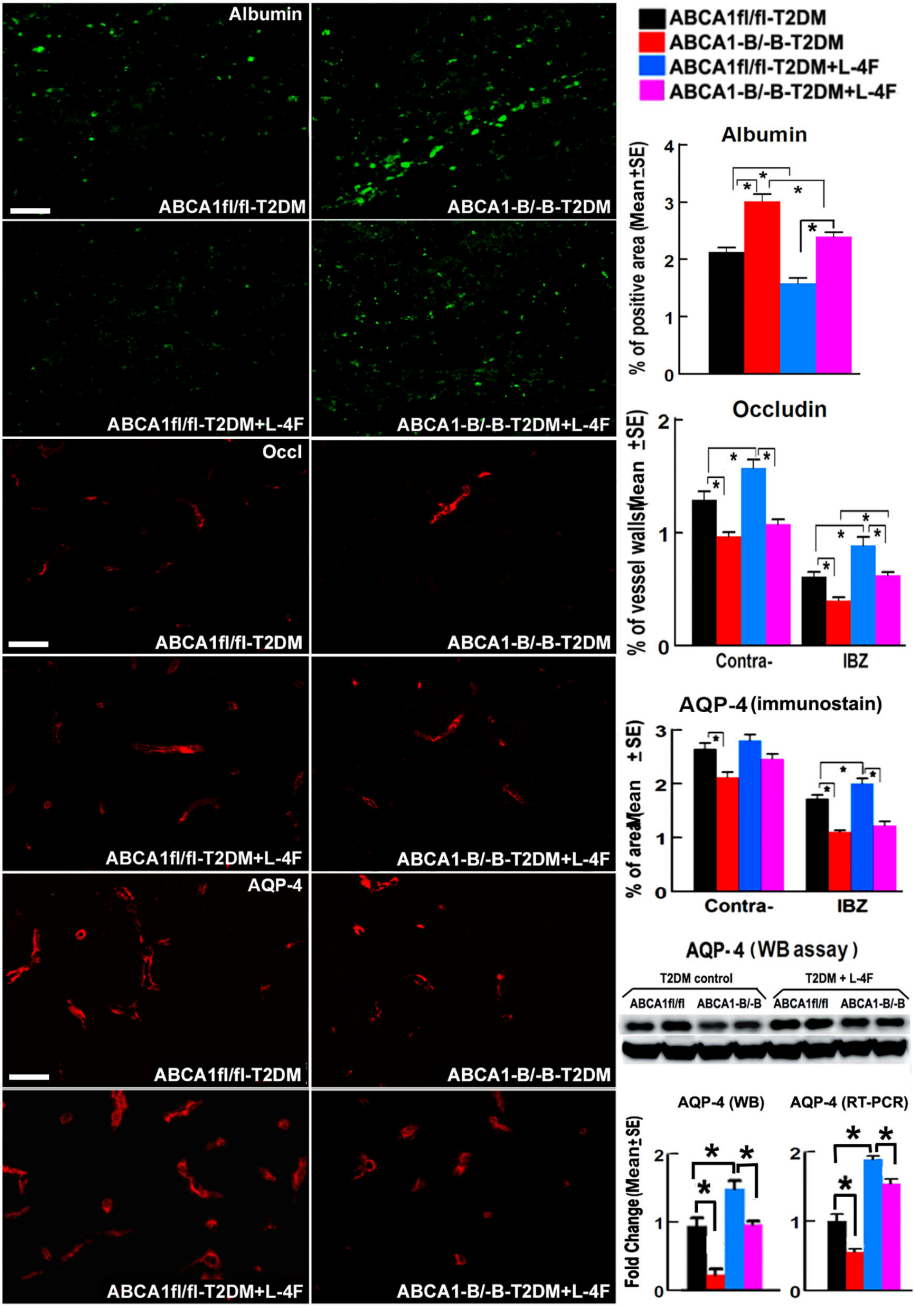
L-4F treatment decreases BBB-leakage (albumin density) but increases tight-junction protein (Occludin) in the ischemic brain of T2DM mice at 21 days after stroke. L-4F treatment did not show an increases AQP-4 expression in astrocyte end-feet measured by immunostaining. However, the WB and RT-PCR data demonstrate that both the protein and mRNA level of AQP-4 in the ischemic brain was increased after 21 days of L-4F treatment. Scale bar = 50 μm; *p < 0.05, *n* = 9/group in immunostaining measurement of Albumin, Occludin and AQP-4, *n* = 6/group in WB and RT-PCR assay.

Figure 2 shows that the density of albumin in the ischemic core area increased in ABCA1^−B/−B^-T2DM stroke mice, while the expression of Occludin and AQP-4 within or around blood vessels in both the contralateral and the IBZ area in the ABCA1^−B/−B^-T2DM stroke mice significantly decreased compared with the ABCA1^fl/fl^-T2DM stroke mice (*p* < 0.05, *n* = 9/group). In addition, WB and RT-PCR assay indicate that the level of AQP-4 in the ischemic brain is decreased in the ABCA1^−B/−B^-T2DM stroke mice (*p* < 0.05, *n* = 6/group). L-4F treatment significantly decreased albumin density indicating improved BBB integrity and increased tight junction protein in the ischemic brain in both ABCA1^−B/−B^ and ABCA1^fl/fl^ T2DM-stroke mice measured by immunostaining (*p* < 0.05, *n* = 9/group). Moreover, L-4F treatment also elevated AQP4 expression in the ischemic brain tissues measured by WB and RT-PCR (*p* < 0.05, *n* = 6/group) even though there is no significant difference by immunostaining analysis.

**Figure 3:**
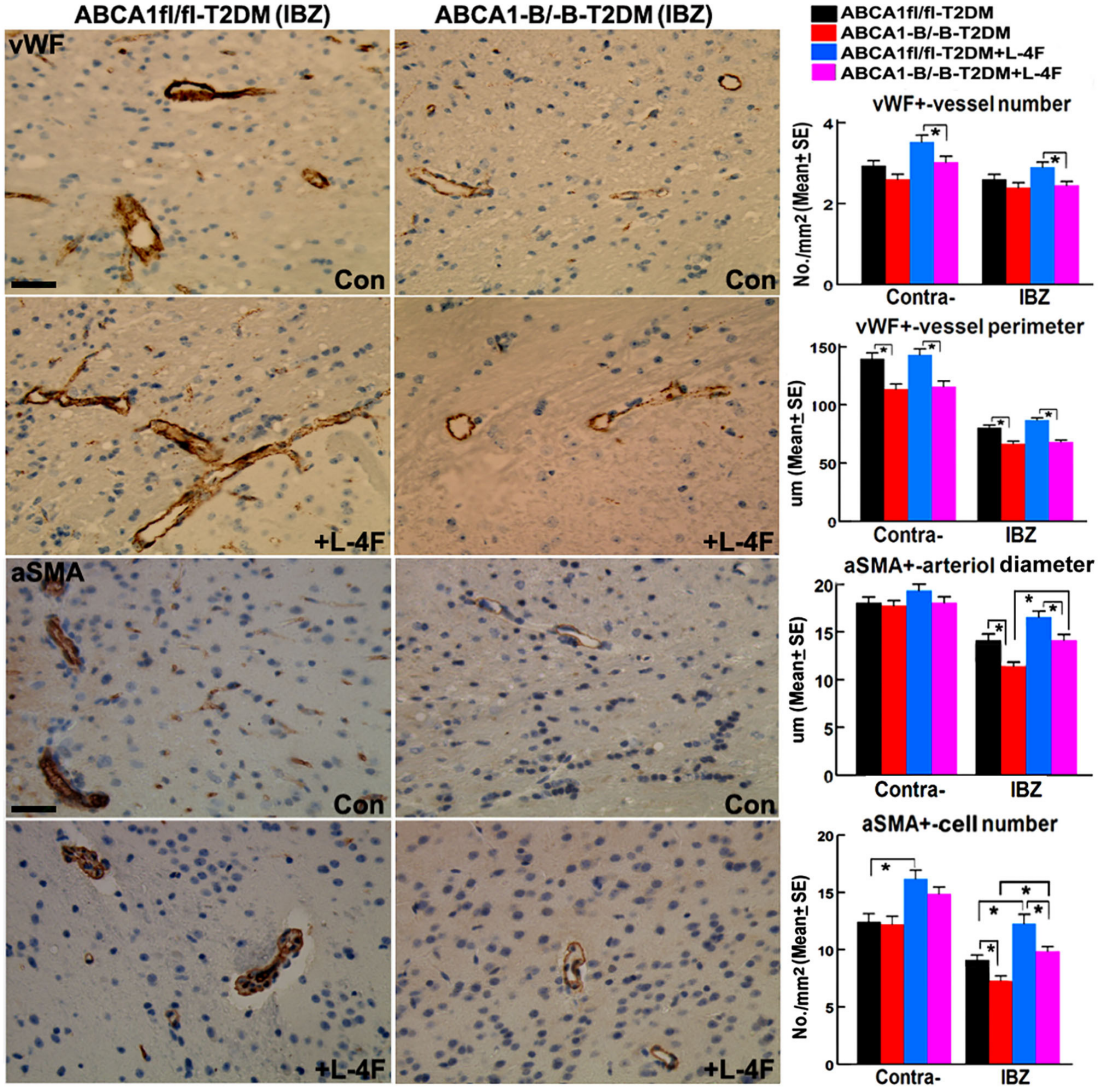
L-4F treatment increases arteriolar diameter in the IBZ of ABCA1^−B/−B^ -stroke mice and increases SMC-number in the IBZ of both ABCA1^fl/fl^ and ABCA1^−B/−B^ T2DM-stroke mice. Scale bar = 50 μm; *p < 0.05, *n* = 9/group.

Figure 3 shows that even though there were no significant differences in the densities of vWF^+^-vessels and αSMA^+^-arterioles in both the contralateral hemisphere and the IBZ, and the diameter of arterioles and the number of αSMA^+^-SMCs in the contralateral hemispheres between the ABCA1^−B/−B^ and ABCA1^fl/fl^ T2DM-stroke mice, ABCA1^−B/−B^-T2DM stroke mice exhibit decreased vessel perimeter in both the contralateral hemisphere and the IBZ, and decreased arteriole diameter and SMC number in the IBZ compared with ABCA1^fl/fl^-T2DM stroke mice (*p* < 0.05, *n* = 9/group). L-4F treatment increased the number of SMCs in both the contralateral hemisphere and IBZ of ABCA1^fl/fl^-T2DM mice and increased SMC number and arteriole diameter in the IBZ of ABCA1^−B/−B^-T2DM stroke mice 21 days after stroke (*p* < 0.05, *n* = 9/groups). These data indicate that T2DM-stroke induces BBB leakage and vascular injury in the ischemic brain, while brain-ABCA1 deficit exacerbates T2DM-stroke induced vascular damage. Administration of L-4F decreased T2DM-stroke induced vascular damage and BBB permeability even in brain-ABCA1-deficient mice.

### L-4F Treatment Increases WM Density and Oligodendrogenesis in the Ischemic Brain of T2DM-Stroke Mice

**Figure 4:**
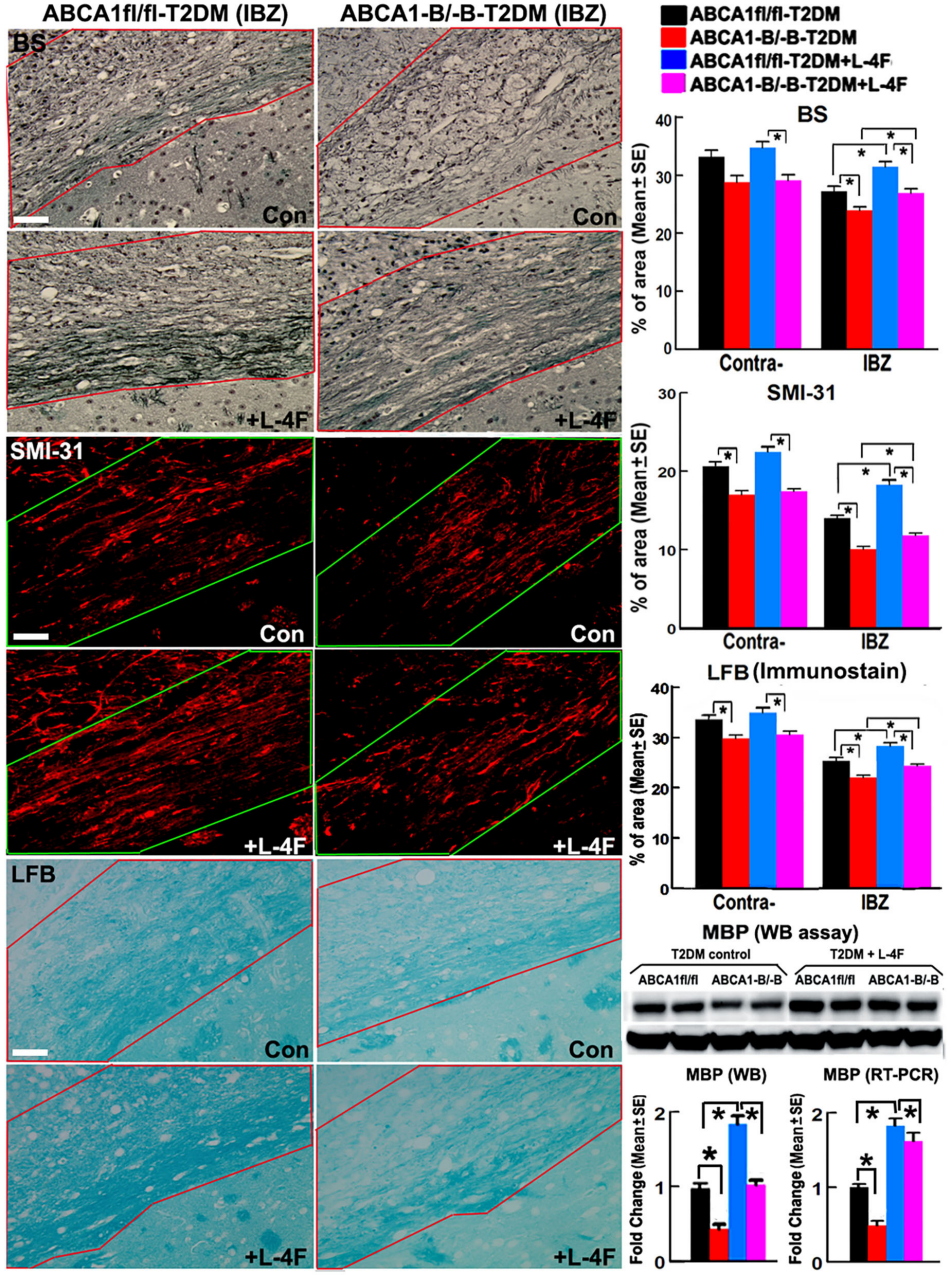
L-4F treatment increases the densities of axon, phospho-neurofilament, and myelin in the WM-bundles in the IBZ, and MBP protein level in the ischemic brain of ABCA1^fl/fl^ and ABCA1^−B/−B^ T2DM mice at 21 days after stroke. Scale bar = 50 μm. *p < 0.05, *n* = 9/group in immunostaining measurement; *n* = 6/group in WB and RT-PCR assay.

**Figure 5:**
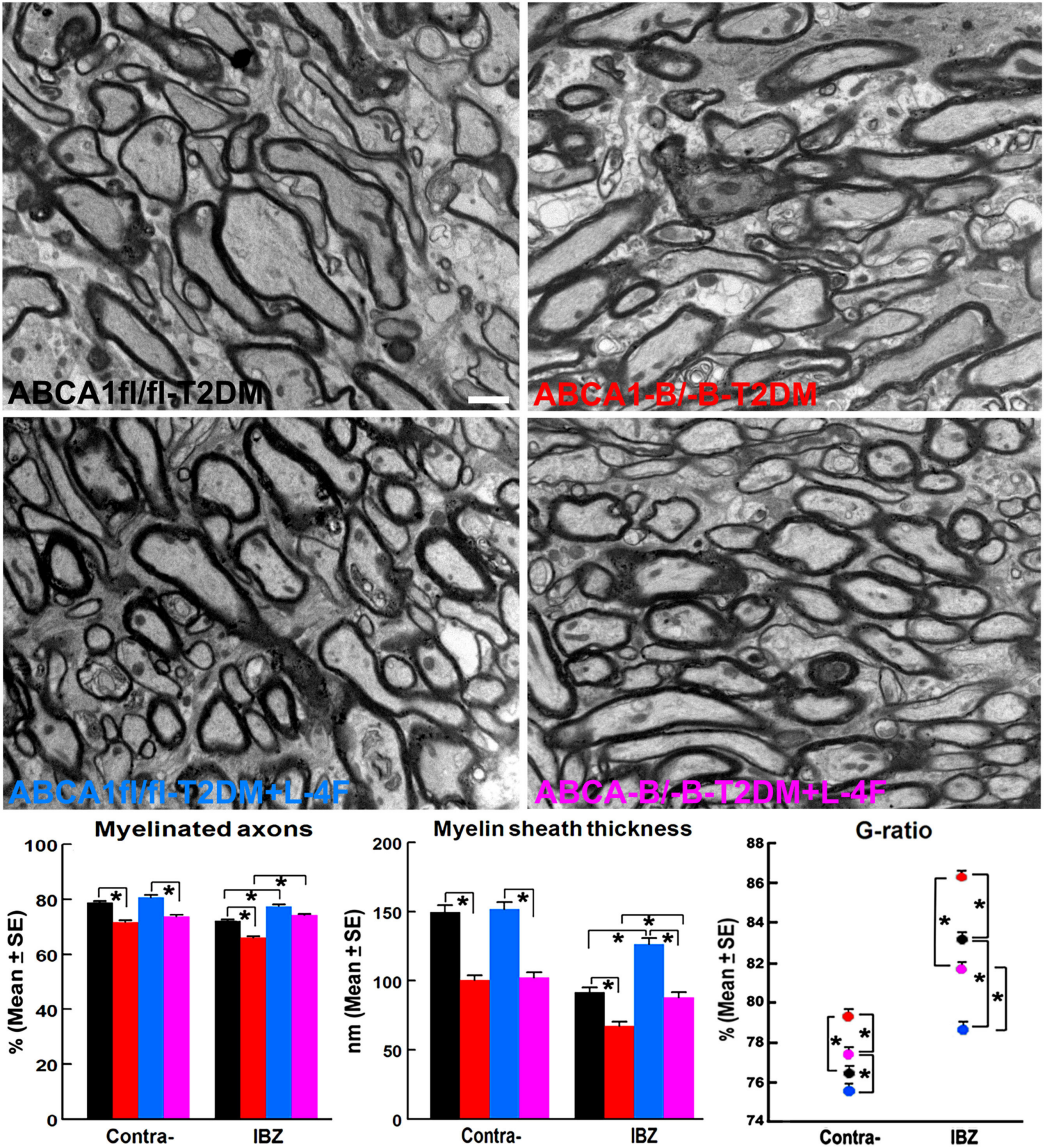
L-4F treatment increases myelination identified by increased density of myelinated-axons, myelin-sheath thickness, and reduced G-ratio in the IBZ of WM-bundles of the ischemic brain at 21 days after stroke in T2DM mice. Scale bar = 1 μm, *p < 0.05, *n* = 6/group.

**Figure 6:**
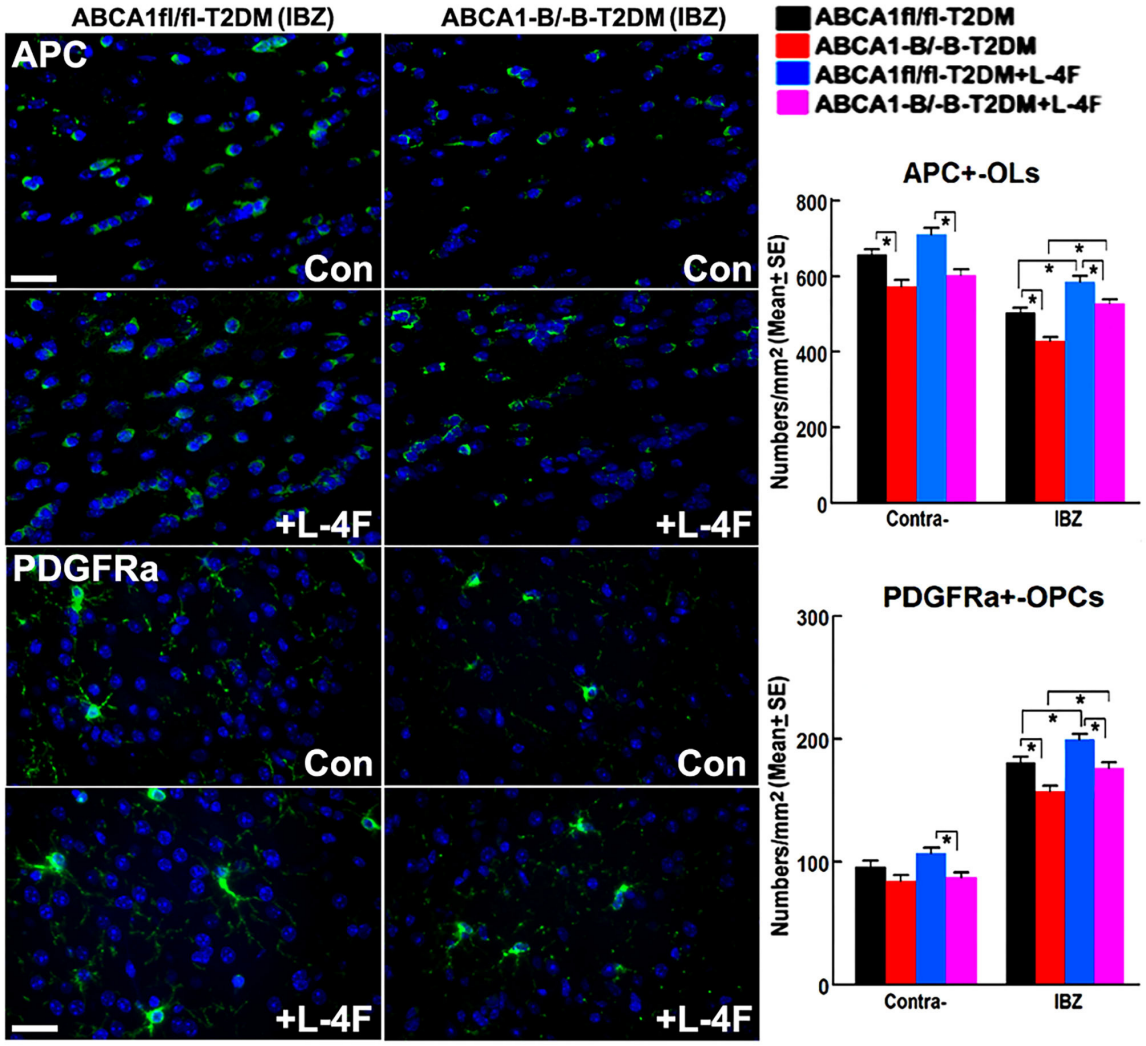
L-4F treatment increases the number of OLs and OPCs in the IBZ of WM-bundles and cortex in the ischemic brain at 21 days after stroke in T2DM mice. Scale bar = 50 μm, *p < 0.05, *n* = 9/group.

The immunohisto-chemical-staining and quantification data show that the ABCA^−B/−B^-T2DM stroke mice exhibit a significant decrease in the densities of axons, phosphorylated-neurofilament, and myelin in the WM-bundles of CC (Figure 4, *p* < 0.05, *n* = 9/group), and WB/RT-PCR assay show that the protein and mRNA level of MBP was decreased in the ischemic brain tissues (Figure 4, *p* < 0.05, *n* = 6/group) at 21 days after dMCAo compared with the ABCA1^fl/fl^-T2DM stroke mice. The analysis from EM images indicates that both the number of myelinated-axons and the thickness of myelin-sheath decreased but G-ratio increased in the WM-bundles of CC in both the contralateral brain and the IBZ of the ischemic brain in the ABCA^−B/−B^-T2DM stroke mice (Figure 5, *p* < 0.05, *n* = 6/group). Moreover, the number of APC^+^-OLs in the CC of the IBZ and the number of PDGFRα^+^-OPCs in the cortex of the IBZ of ABCA^−B/−B^-T2DM stroke mice were also reduced (Figure 6, *p* < 0.05, *n* = 9/group). L-4F treatment significantly decreased G-ratio, increased the axon density, phosphorylated-neurofilaments, myelin density, number of myelinated-axons, myelin-sheath thickness, and the number of OLs and OPCs in the IBZ (*p* < 0.05, *n* = 9/group) and also the protein/mRNA levels of MBP in the ischemic brain tissues (*p* < 0.05, *n* = 6/group) in both ABCA1^fl/fl^-T2DM and ABCA^−B/−B^-T2DM stroke mice when compared with the vehicle-control group, respectively. These data indicate that L-4F treatment promotes WM remodeling and oligodendrogenesis in the ischemic brain after stroke in T2DM mice.

### Identification of Localization of FITC-Labeled D-4F After Treatment

**Figure 7:**
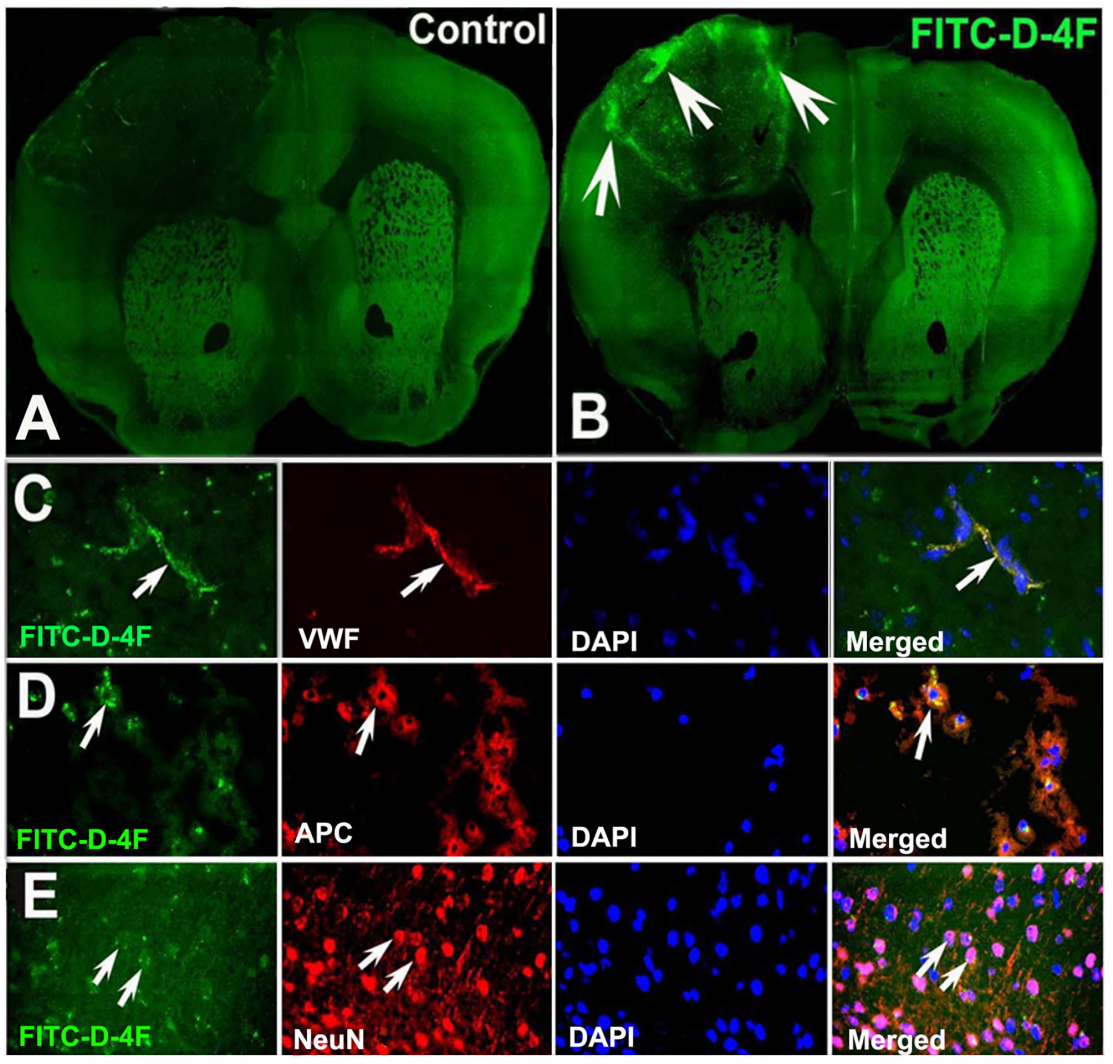
D-4F can pass through the BBB and enter into the ischemic brain, and was co-localized with vWF^+^-vessels, APC^+^-OLs, and NeuN^+^-neurons in the IBZ of the ischemic brain of T2DM-stroke mice. The confocal image of mouse brain in control **(A)** and FITC-D-4F **(B)**. D-4F was co-localized with vascular vessels **(C)**, OLs **(D)** and neurons **(E)**, respectively.

Figure 7 shows that the FITC-labeled D-4F can pass through the BBB to enter the ischemic lesion area and was primarily present in vWF^+^-vessels and APC^+^-OLs and NeuN^+^-neurons in the ischemic brain.

### L-4F Treatment Reduces Macrophage Infiltration and Decreases Inflammation in the Ischemic Brain in T2DM-Stroke Mice

**Figure 8:**
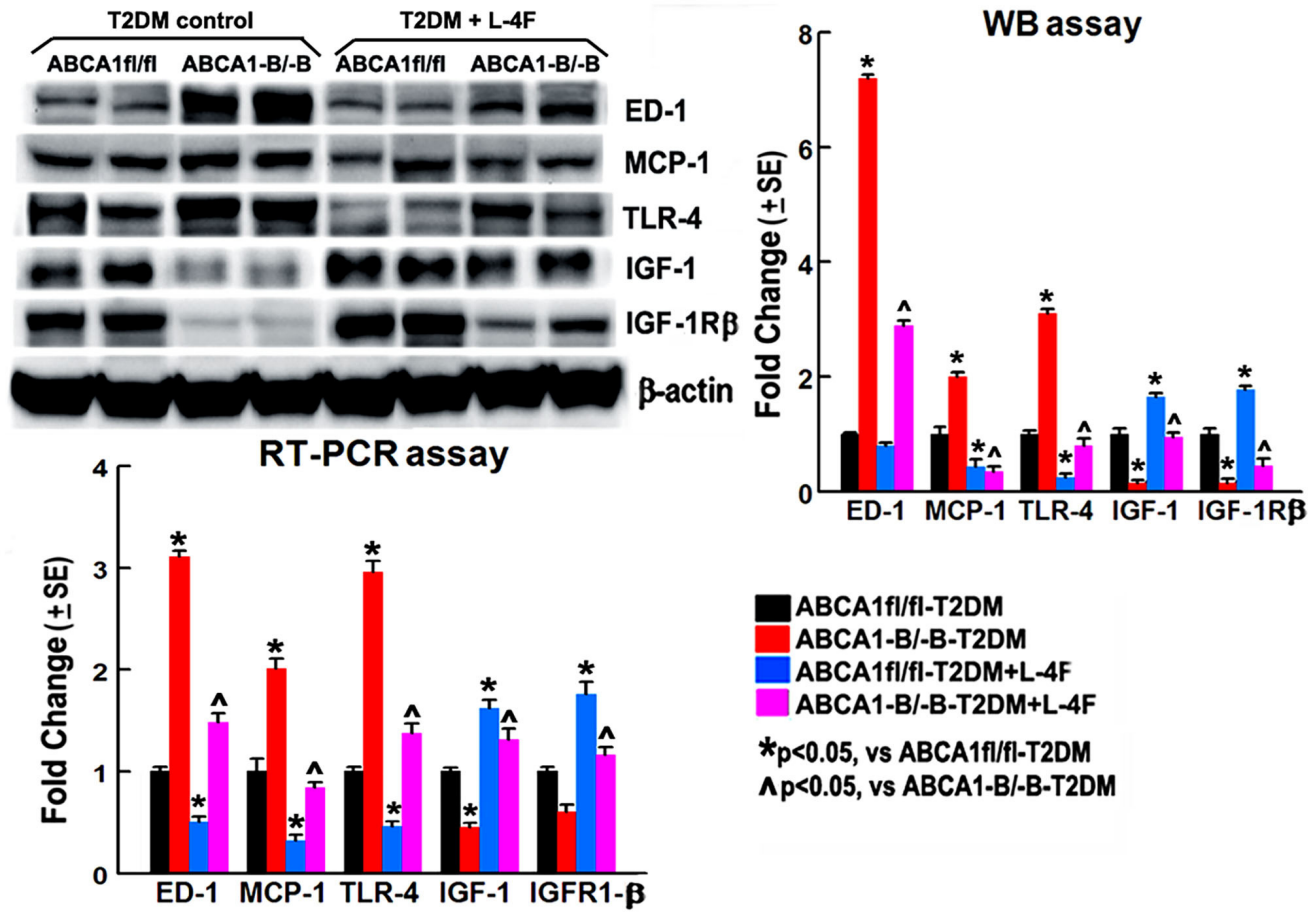
L-4F treatment decreases ED1, MCP-1, and TLR-4 but increases IGF-1 and IFGR-1β in the ischemic brain in both ABCA1^fl/fl^ and ABCA1^−B/−B^ T2DM mice at 21 days after stroke measured by WB or RT-PCR. *n* = 6/group.

To investigate the mechanism underlying L-4F treatment-induced neurorestoration in T2DM-stroke, the macrophage/microglial and monocyte infiltration was measured using the levels of ED-1 and MCP-1, and the inflammatory factor TLR-4 and anti-inflammatory factors IGF-1 and IGF-1Rβ were measured using WB and RT-PCR assay. Figure 8 shows that the ABCA^−B/−B^-T2DM stroke mice exhibit an increased level of protein and mRNA of ED-1, MCP-1, and TLR-4, whereas a lower level of IGF-1/IGF-1Rβ in the ischemic brain tissues when compared that with the ABCA1^fl/fl^-T2DM stroke mice at 21 days after dMCAo (*p* < 0.05, *n* = 6/group). L-4F treatment significantly decreases ED-1, MCP-1, and TLR-4 expression and increases IGF-1 and IGF-1Rβ levels in the ischemic brain compared with the vehicle-control group, respectively (*p* < 0.05, *n* = 6/group). These data indicate that post-stroke administration of L-4F in T2DM-stroke mice inhibits neuroinflammation which may contribute to the L-4F treatment-induced neurorestoration after brain injury.

## Discussion

The BBB contains vascular endothelial cells (ECs), pericytes, tight junction, and astrocyte end-feet and interacts directly with astrocytes and neurons in the neurovascular niche (65–67). The BBB acts selectively as a transport interface and therapeutic agents must pass the BBB to provide successful treatment of brain injury or disease (67). Using commercially available FITC-labeled D-4F, we demonstrated that D-4F/L-4F can pass through the BBB to enter brain tissue and cells including ECs, OLs, and neurons. Long-term endothelial dysfunction and impaired vasodilation, as well as increased vasogenic edema and BBB leakage in the ischemic brain, are highly associated with T2DM as a predictor for secondary cerebrovascular events such as hemorrhage (68). Neurovascular dysfunction triggers and exacerbates WM damage which in turn hinders neurological functional recovery, and leads to an enlarged infarct volume after stroke in both the experimental T2DM-stroke model and T2DM-stroke patients (51, 64, 69–73). In this study, we demonstrate that L-4F treatment not only has beneficial effects on BBB integrity (identified by decreased albumin infiltration, increased tight junction protein expression, and AQP-4 expression in astrocyte end-feet), but also on neurovascular remodeling (identified by increased SMC number and enhanced vasodilation), WM remodeling (identified by increased WM density and myelination), and oligodendrogenesis (identified by the increased number of OLs and OPCs) in the ischemic brains in the middle-aged wild-type (ABCA^fl/fl^) T2DM-stroke mice. These results are consistent with our previous findings that treatment of db/db-T2DM stroke mice with L-4F reduced hemorrhage, infarct volume, mortality, BBB leakage and WM damage, and increased cerebral arteriole diameter and SMC number in the ischemic brain 4 days after stroke (46). We also found previously that *in vitro*, L-4F treatment does not increase angiogenesis in mouse-brain ECs cultured in high-glucose media, but increases primary artery explant cell migration after stroke-induced injury and enhances neurite and axonal outgrowth in primary cortical neurons subjected to oxygen-glucose deprivation or high glucose (46). D-4F treatment of T1DM-stroke rats also increases tight junction protein expression and decreases BBB leakage, WM damage, and pro-inflammatory factors, while increasing anti-inflammatory M2 macrophage polarization in the ischemic brain 7 days after stroke (45). L-4F decreases markers of plasma oxidation and promotes EC migration and EC-healing of carotid arterial injuries in HFD-fed mice (74). L-4F-treated mice exhibit reduced serum levels of oxidized phospholipids and increased mean αSMA^+^-area in the aortic lesion in a murine lupus model of accelerated atherosclerosis (75).

In the peripheral blood system, ApoA-I is a major apolipoprotein involved in HDL formation and is highly influenced by ABCA1, and ABCA1 is usually found in tissue macrophages and activated monocytes (39, 76–80). In the CNS, HDL synthesis uses ApoE as the predominant apolipoprotein regulated by ABCA1 *via* facilitating cholesterol and phospholipid efflux to exogenous ApoE (81–83), and ABCA1 is also highly expressed in neurons, astrocytes, OLs, and microglia (26, 29, 84–87). D-4F promotes cholesterol efflux from macrophages *via* ABCA1 (42). In this study, L-4F treatment increases blood HDL in ABCA^fl/fl^-T2DM stroke mice. Since L-4F can cross the BBB and reach the CNS, to further elucidate whether L-4F treatment-induced neurorestorative effect on T2DM-stroke is mediated by ABCA1 and ApoE signaling pathway, ABCA^−B/−B^ mice were employed. These mice were generated by crossing loxP-flanked (floxed) ABCA1 mice with nestin-cre mice, by which the ABCA1 gene is knocked out from all nestin-linage cells (neural stem cells) in the brain including neurons, OLs, and astrocytes, and these mice exhibit reduced brain ABCA1 and ApoE and HDL content, and reduced level of ABCA1, ApoE and HDL was also found in a primary cortical neuron or OPC cultures (17, 29, 34–36, 47). Our results indicate that inhibition of brain ABCA1 does not attenuate the neurorestorative benefits induced by L-4F treatment in ABCA^−B/−B^-T2DM stroke mice. D-4F is capable of forming HDL-like particles and delivering cholesterol to the liver cells selectively through the scavenger receptor class B type I (SR-BI) and enhances the cholesterol delivery by native HDL (40). D-4F treatment increases Lecithin cholesterol acyltransferase activity, increases HDL levels and cholesterol efflux from macrophages, decreases inflammation and oxidative stress, improves renal histological pathogenesis, and causes lesion regression in ApoE(-/-) mice (38, 88–90). D-4F also improves arterial vasoreactivity and arterial wall thickness in hypercholesterolemic LDLR(-/-) mice and LDLR(-/-) and ApoA-I(-/-) double-knockout mice which independent of ApoA-I (59, 91). Administration of L-4F increases HDL and ApoA-I concentration in the plasma, decreases albuminuria and stimulates cholesterol efflux and related proteins expressions, and reduces atherosclerotic lesions in ApoE (-/-) mice (39, 43, 92). GW3965, an LXR agonist increases ApoA-I protein levels in the CNS independent of ABCA1, which suggests that ApoA-I may be regulated by distinct mechanisms on either side of the BBB and that ApoA-I may serve to integrate peripheral and CNS lipid metabolism (27). This study along with previous studies suggests that L-4F derived therapeutic effects in brain rewiring after stroke injury may, at least partially, be independent of the ABCA1, ApoE, or ApoA-I.

Compared with non-DM stroke, T2DM stroke have chronic states of oxidative stress and inflammation, which is highly associated with extensive microvascular and WM damage (14, 50, 64, 93–97). Some studies report that D-4F and L-4F have no effect on blood lipid, T-CH, or HDL levels but improve HDL function in anti-oxidation and anti-inflammation in both the peripheral and cerebral vascular systems (37, 38, 56, 59–63, 74, 93, 98–102), which implies that D-4F and L-4F, similar to HDL, have neuroprotective and neurorestorative potential in atherosclerosis (37, 92, 101, 103–105), diabetes (101, 106, 107), and ischemic stroke (45, 46). L-4F has the ability to preferentially bind to proinflammatory oxidized lipids and decrease serum and endothelium oxidized-LDL levels and improve vasodilation by stimulation of the arterial wall cells including ECs and SMCs in both wild-type and LDLR(-/-) mice fed with or without HFD food (56–58, 108–111). Administration of D-4F improved the migration of ECs and angiogenesis, alleviated oxidative stress (107), and decreased circulating EC sloughing, superoxide anion levels, and vasoconstriction in diabetic rats, which are associated with an increase in antioxidant proteins, HO-1 and EC-SOD (93). D-4F and L-4F have the ability to inhibit LDL-induced monocyte chemotactic activity *via* inhibiting MCP-1, a key chemokine that regulates migration and infiltration of monocytes and macrophages, production in cultures of human aortic ECs (100, 112). D-4F suppressed IL-4-induced macrophage alternative activation and pro-fibrotic TGF-β1 expression (113) and L-4F-reduced vascular cell adhesion molecule-1 expression in lipopolysaccharide (LPS)-induced inflammatory responses (114). D-4F treatment evokes a vascular protective role in LPS-induced acute lung injury by improving the endothelial progenitor cell (EPC) numbers, differentiation, and function, and decreasing plasma levels of the pro-inflammatory mediators such as TNF-α and ET-1 partially *via* the PI3K/AKT/eNOS signaling pathway (115). D-4F treatment also reduces infiltration of macrophages in diabetic ApoE-/- mice (101), and also decreases arterial macrophage traffic and inflammatory factors such as IL-1β, IFN-⋎, and TNFα on HFD-fed mice or rabbits (106, 116, 117), In thisstudy, L-4F treatment decreased the infiltration of M1-macrophages, MCP-1, and TLR-4 (a key inflammatory factor expressed in M1-macrophages whose activation leads to activation of the intracellular signaling pathway NFκB and inflammatory cytokine production), while increasing IGF-1 and IGF-1Rβ in the ischemic brain in both ABCA^fl/fl^ and ABCA^−B/−B^ mice. These results are consistent with our previous findings that D-4F treatment of T1DM rats decreases MMP9 and proinflammatory mediators TNFα, TLR-4 and increases anti-inflammatory M2-macrophage polarization (45). Administration of L-4F to db/db T2DM stroke mice mitigated macrophage infiltration and reduced TNFα (46) in the ischemic brain. IGF1 is involved in neurogenesis, oligodendrogenesis, and myelination (118), and reduces stroke-induced BBB damage and sustained antiinflammation in the brain (119). IGF1 also decreases cholesterol efflux *via* ABCA1 and SR-BI expression (120). Similarly, in T2DM patients L-4F treatment restored the HDL anti-inflammatory index in diabetic plasma samples (121).

In this study, L-4F-treated T2DM-stroke animals also exhibit decreased blood glucose level, which is consistent with our previous results that L-4F decreased high-mobility group box-1 (HMGB-1), advanced glycation end-product receptor (RAGE), and plasminogen activator inhibitor-1 (PAI-1) in the ischemic brain in T2DM-stroke mice (46). D-4F treatment also increased blood glucose clearance and improve insulin tolerance in HFD-fed mice (106) and D-4F treatment ameliorated disordered glycolysis impaired by ox-LDL (107). Whether L-4F treatment-induced neurorestorative benefits are due to blood glucose regulation warrants future investigation.

### Summary

In this study, we demonstrate that L-4F can pass through the BBB and has neurorestorative benefits such as promoting neurovascular and WM remodeling and oligodendrogenesis in the ischemic brain as well as improvement of neurological functional recovery in T2DM-stroke mice. L-4F-treated T2DM-stroke mice exhibit suppressed neuroinflammation in the ischemic brain of both ABCA1^−B/−B^ and ABCA1^fl/fl^ T2DM stroke mice. These findings indicate that post-stroke administration of L-4F may provide a potential strategy for neurorestoration following stroke injury in the T2DM population, and reducing neuroinflammation in the injured brain may contribute to the neurorestorative effects of L-4F at least partially independent of the ABCA1 signaling pathway.

### Limitation

In this study, all brain tissue samples were collected 21 days after stroke. Tissue sampling at this late time point enables the study of long-term vascular and WM remodeling effects in the brain, however, from this delayed sampling we are unable to glean information of BBB leakage and inflammatory responses at the acute phase of stroke recovery, which warrants future investigation.

## Data Availability Statement

The raw data supporting the conclusions of this article will be made available by the authors, without undue reservation.

## Ethics Statement

The animal study was reviewed and approved by Institute of Animal Care and Use Committee of the Henry Ford Health System.

## Author Contributions

XC and QJ conceived and designed this study. MZ, RL, AZ, YQ, JL-W, PV, and XC performed mouse experiments, acquisition of data, and statistical analysis. XC wrote and prepared this manuscript. PV and MC made critical revisions of the manuscript for intellectual content. All authors read and approved the final manuscript.

## Funding

This study was supported by the National Institute of Neurological Disorders and Stroke grant to QJ and XC (R01NS097747).

## Conflict of Interest

The authors declare that the research was conducted in the absence of any commercial or financial relationships that could be construed as a potential conflict of interest.

## Publisher's Note

All claims expressed in this article are solely those of the authors and do not necessarily represent those of their affiliated organizations, or those of the publisher, the editors and the reviewers. Any product that may be evaluated in this article, or claim that may be made by its manufacturer, is not guaranteed or endorsed by the publisher.
